# Radiographic bone level and soft tissue dimensional changes following explantation of implants affected by peri‐implantitis: A retrospective exploratory evaluation

**DOI:** 10.1002/cre2.802

**Published:** 2023-10-31

**Authors:** Giovanni Serino, Masahiro Wada, Tomoaki Mameno, Andreas Stavropoulos

**Affiliations:** ^1^ Clinic of Periodontology Public Dental Service Borås Region Västra Götaland Sweden; ^2^ Research and Development Unit Public Dental Service Borås Region Västra Götaland Sweden; ^3^ Department of Removable Prosthodontics and Gerodontology, Graduated School of Dentistry Osaka University Osaka Japan; ^4^ Department of Periodontology, Faculty of Odontologi Malmö University Malmö Sweden; ^5^ Division of Conservative Dentistry and Periodontology, University Clinic of Dentistry Medical University of Vienna Vienna Austria; ^6^ Department of Periodontology, School of Dental Medicine University of Bern Bern Switzerland

**Keywords:** alveolar socket, dental implants, implant explantation, peri‐implantitis

## Abstract

**Background:**

While the dimensional alteration of alveolar bone following tooth extraction have been extensively descripted in the literature, no information is available regarding potential hard and soft tissues changes following implant explantation.

**Aim:**

To evaluate the radiographic bone healing and the horizontal and vertical soft tissue dimensional alterations at implant extraction alveoli, 6 months following implant explantation.

**Material and Methods:**

Data from 31 patients scheduled for extraction of one implant with persisting peri‐implantitis despite treatment were analysed. Bone crest level changes and the extent of bone healing at the apical aspect of the implant socket were assessed on the radiographs prior and 6 months following explantation. Regression analyses assessed the impact of various predictors (e.g., bone crest level, presence/absence of buccal bone) on bone level changes. Fisher's exact probability test was applied to assess the difference in probability to have mucosa recession of ≥2 mm in the presence or absence of alveolar buccal bone.

**Results:**

A vertical bone loss of 0.8 mm (standard deviation [*SD*] = 1.3) of the peri‐implant bone crest and a gain of 0.8 mm (*SD* = 1.1) from the bottom of the peri‐implant defect were recorded. Complete healing was noted in the intact implant extraction socket (i.e., the part of the implant not affected by peri‐implantitis). A reduction of 0.4 mm (*SD* = 0.7) of the alveolar mucosa height was recorded in concomitant with a decrease of 0.7 mm (*SD* = 0.8) of the mucosa width. These alterations were more pronounced in the absence of the alveolar buccal bone.

**Conclusion:**

The results of the present explorative study indicated a decrease in the height and width of the alveolar soft and hard tissues following explantation of peri‐implantitis affected implants, and these changes were more pronounced in the absence of the buccal bone wall. Nevertheless, the apical portion of the implant alveolus (the intact implant socket) tend to heal with no further bone loss.

## 1 INTRODUCTION

Tooth extraction sockets heal usually uneventfully with bone tissue 1–2 months following tooth removal (Amler et al., 1960; Evian et al., 1981), although complete healing including cortication of the sockets takes often about 9–12 months (Bertl et al., 2018). Healing usually occurs with substantial reduction of the original height and width of the alveolar bone and with the remodeling of overlying soft tissue. A systematic review of postextraction alveolar hard and soft tissue dimensional changes in humans concluded that a horizontal bone loss of 26%–63% and a vertical bone loss of 11%–22% after 6 months following tooth extraction could be expected (Tan et al., 2012). Different factors may influence the erratic healing of an extraction socket, one of those is the loss of buccal bone (Kim et al., 2014).

Dental implants are widely used to restore missing teeth. However, implants may need to be removed in case of complications (Esposito et al., 2005), e.g., peri‐implantitis, implant fracture etc. Different techniques have been used to explant dental implants. Those techniques include block resection, buccal bone osteotomy, trephine osteotomy, circumferential osteotomy with piezo‐surgery; all of those techniques, however, require additional removal of bone.

This may render the implant extraction sites unsuitable for the insertion of a new implant and/or compromise the aesthetics due to substantial hard and soft tissue dimensional alteration.

Recently a new technique to remove implants has been introduced to preserve the residual bone surrounding the explanted implants (Anitua et al., 2016). This technique is based on the application of counter‐torque to break the implant‐bone attachment. Up to now, the extent of bone and soft tissue dimensional changes following implant extraction by means of counter‐torque device has not yet been evaluated.

The aim of this explorative study was to evaluate the radiographic bone healing and the horizontal and vertical soft tissue dimensional alterations at implant extraction alveoli 6 months following implants removal.

## 2 MATERIALS AND METHODS

### 2.1 Patient recruitment

The data of this retrospective clinical evaluation were collected from the records and radiographs of patients treated for peri‐implantitis at the Specialist Clinic in Periodontology, Södra Älvsborgs Hospital, Borås, Sweden, between 2018 and 2020. When the patients were informed about the treatment, they were also informed that their data would be later used anonymously for statistical analyses. Appropriate informed consent was obtained. Ethics approval was obtained from the ethical review authority of Sweden (document no. 2021‐05819‐01).

#### 2.1.1 Inclusion criteria

All patients who underwent implant explantation between 2018 and 2020. The patients were attending supportive peri‐implant treatment for at least 5 years following surgical treatment of peri‐implantitis and had at least one implant with persisting clinical signs of peri‐implantitis following peri‐implant surgery (i.e., probing pocket depth ≥6 mm, bleeding on probing and/or suppuration) giving discomfort to the patients (pain, swelling and/or suppuration). Consequently, those implants were scheduled for explantation.

#### 2.1.2 Exclusion criteria

Implants presenting mobility (i.e, complete loss of osseointegration) or with the remaining osseointegrated portion of the implant being less than 4 mm (as judged in peri‐apical radiographs).

### 2.2 Treatment

**Figure 1:**
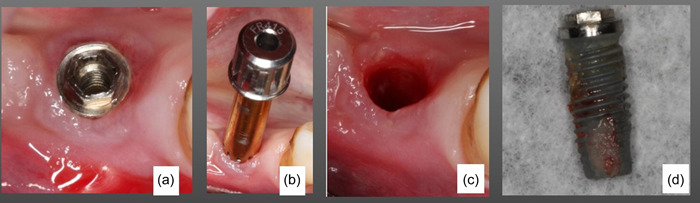
From left (a) implant before explantation, (b) the fixture‐remover is screwed over the fixture‐remover screw counterclockwise until reach the neck of the implants, (c) inspection of the alveola after implant removal, (d) The explanted implant.

Following the removal of the prosthesis and of the abutment (when present), the distance between a reference point at the opposite jaw and the mid‐buccal mucosa margin at the implant scheduled for the extraction was registered, using a manual periodontal probe (Hu‐Friedy PCP15 periodontal probe Hu‐Friedy). The buccal‐lingual/palatal width of the alveolar process, including the mucosa was also measured likewise. Following those measurements, local anaesthesia was infiltrated in the area around the implant that should be extracted. If the implant was localized in a sub‐mucosal position a punch was used to remove the overlying soft tissue to localize the neck of the implant. The implants were removed using an Implant Removal Kit® (Biomet 3I) following the recommendations of the manufacturer: first a “Fixture Remover Screws” (compatible for the various implant systems) was screwed into the implant using a hex driver mounted on a torque wrench turned clockwise at 50–60 Ncm. Then a “Fixture‐remover” fitting the implant diameter was screwed by hand over the “Fixture‐Remover Screws” counter‐clockwise until reach the neck of the implant. Finally, the removal torque was exerted by the wrench in counter‐clockwise direction, maintaining a perpendicular position, at 200–450 Ncm (Figure 1a,b).

Following implant removal, the postextraction socket was thoroughly debrided to remove any remaining granulation tissue. The extraction socket was carefully examined to identify the status of the residual bony walls (Figure 1c,d). The flaps were then sutured, with no attempt to achieve primary closure of the surgical wound and the prosthetic supra‐structures were reconnected when possible. The patients were instructed to rinse with 0.12% chlorhexidine di‐gluconate twice daily until the time of suture removal at 14 days; at this point, none of the patients reported clinical complications following implant explantation. The patients were recalled at 6 months for re‐evaluation. At this time, the prostheses were removed and the measurements of the height and buccal‐lingual/palatal width of the mucosa overlying the explantation site were repeated in a manner similar to the one previously described. The clinical measurements and treatments were performed by an experienced periodontist (GS) while the radiographic measurements were performed by experienced prosthodontics (MW and TM), not involved in the treatment.

### 2.3 Radiographic examinations

Two diagnostic digital intraoral radiographs (prior implant removal and at 6 months following explanation) were taken for each patient using a parallel technique with film holders. On those radiographs, measurements were done using a program for digital radiographic images (Planmeca Romexis) with ×10 magnifying power and a precision of 0.1 mm.

**Figure 2:**
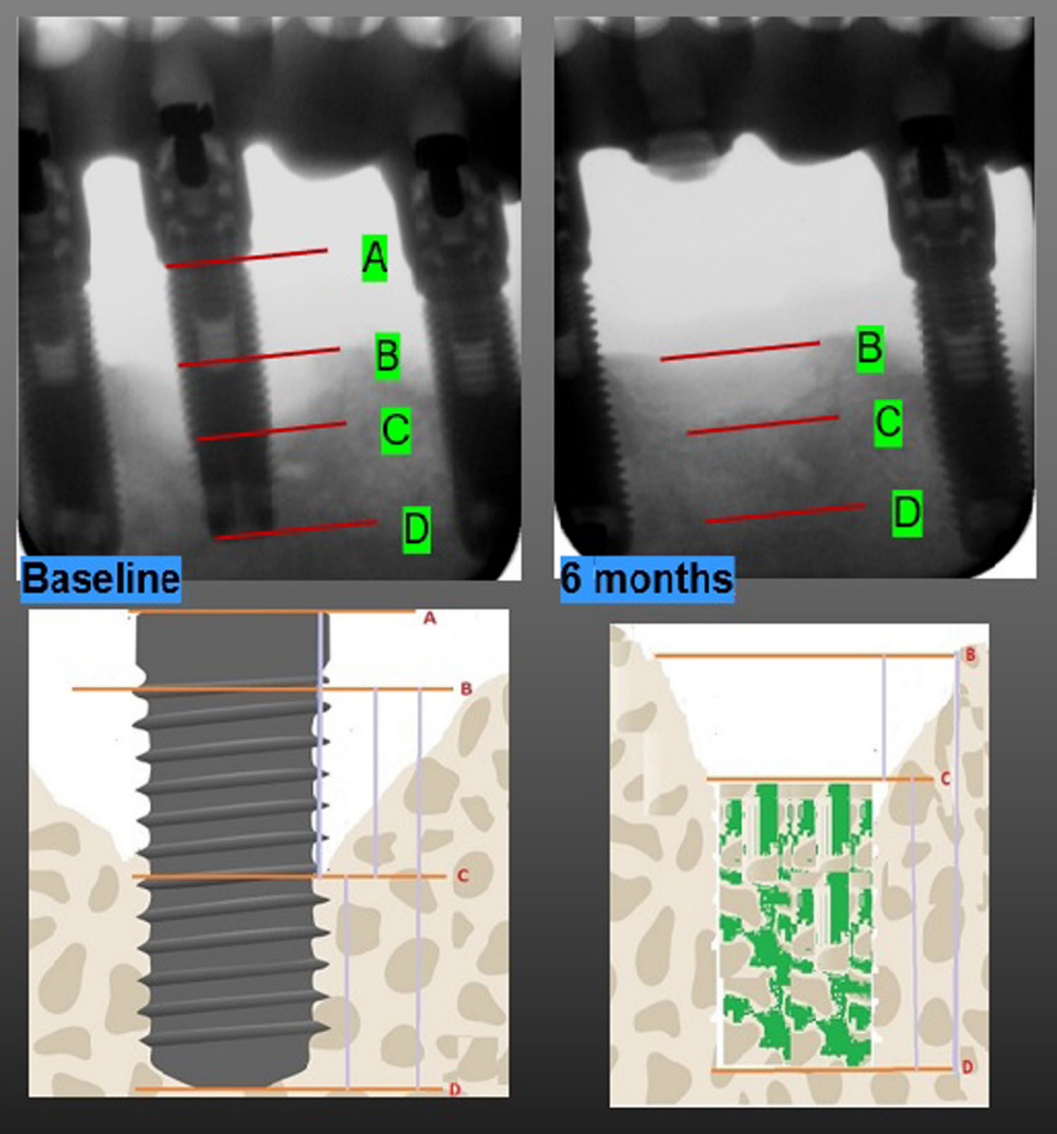
References points for the measurements before and after implants removal.

The following references points were identified on the radiographs (Figure 2)

A: neck of the implants. Depending on the implant system, was either from the fixture‐abutment junction (for the bone level implants) or the shoulder of the implants (for the tissue level implants).

B: the level of the bone crest mesially and distally to the implant on the baseline X‐ray and the bone crest mesially and distally at the implant extraction socket on the 6‐month X‐ray.

C: the bottom of the pier‐implant bone defect at the mesial and distal aspect of the implant on the baseline X‐ray or the bottom of the residual extraction socket on the 6‐month X‐ray.

**Figure 3:**
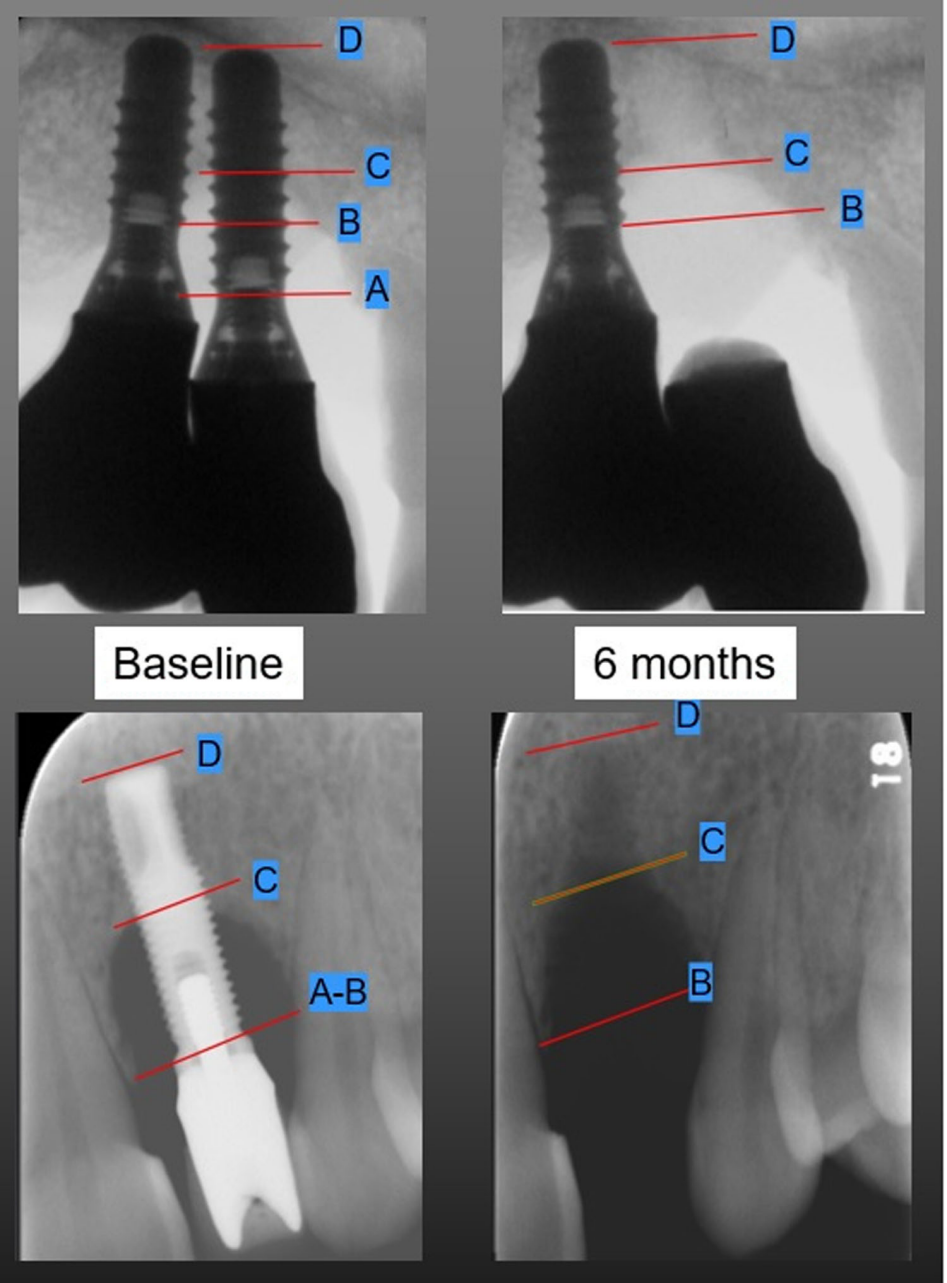
Radiographs before and after implant explantation: It is possible to note the different degree of mineralization in the apical portion between the alveola bony walls and the new formed bone.

D: the apex of the implant on the baseline X‐ray or the apical level of the “intact” implant socket on the 6‐month X‐ray, visible by the different degree of bone mineralization (Figure 3).

The following linear measurements between two points were evaluated:

A‐C: *Exposed implant length* (distance from the neck or the implant and the bottom of the peri‐implant bone defect at the mesial and distal aspect of the implant).

B‐C: *intrabony defect* depth (the distance between the level of the bone crest and the bottom of the peri‐implant bone defect at the mesial and distal aspect of the implant on the baseline X‐ray, or the bottom of the residual extraction socket on the 6‐month X‐ray).

B‐D: *Bone crest level* (distance between the level of the bone crest mesial and distal of the implant and the apex of the implants on the baseline X‐ray or the apical level of the “intact” implant socket on the 6‐month X‐ray).

D‐C: *Intact/healed implant socket length* (the distance between the apex of the implant and the bottom of the peri‐implant bone defect at the mesial and distal aspect of the implant on the baseline X‐ray or the distance between the apical level of the intact implant and the bottom of the residual implant extraction socket on the 6‐month X‐ray).

Before the radiographic measurements, intraobserver error and interobserver error were calculated based on 15 radiographs, and no significant differences in intraobserver error (correlation coefficient: 0.996, 95% confidence interval [CI]: 0.982–1.000) and interobserver error (correlation coefficient: 0.994, 95% CI: 0.985–0.99) were confirmed. The mean (standard deviation [*SD*]) of the difference between the double measurements was 0.11 (0.09) for TM and 0.05 (0.05) for MW.

### 2.4 Data analysis

Mean value, 95% CI, SD, median, interquartile range, minimum and maximum values were used for descriptive statistics.

The outcome variables of interest of this evaluation were:

(1) Changes in bone crest level (i.e., B‐D)
(2) Change in the intact/healed implant socket length (i.e., D‐C)
(3) Changes of the height and width of the alveola mucosa before‐ and 6 months after implant removal.

Wilcoxon signed‐rank test was applied for intraindividual differences between the linear measurements before and after the implant removal. Multilevel linear regression analysis by generalized estimating equations was performed with (1) the bone crest level change (B‐D) as dependent variable and the following independent predictors: bone crest level B‐C, % of bone loss/implant length, presence/absence of buccal bone, implant diameter; (2) the intact/healed implant socket change (D‐C) as dependent variable and the following independent variables: bone crest level (B‐C), % of bone loss/implant length, presence/absence of buccal bone, implant diameter. The side of the implant being mostly exposed (i.e., with the largest A‐C length) was used for the analyses. Fisher's exact probability test was applied to assess the difference in probability to have mucosal recession of ≥2 mm in the presence or absence of alveolar buccal bone. A *p* value of <.05 was considered statistically significant.

## 3 RESULTS

Out of the 37 patients having an implant explanted between 2018 and 2020, three were excluded because the remaining radiographic integrated aspect of the implants (i.e., D‐C) was less than 4 mm and three patients did not come to the 6‐month evaluation because of reasons unrelated to the therapy. Data from 31 patients were finally analysed.

### 3.1 Baseline clinical and radiographical data

Out of the 31 patients included in the data analysis, 20 were females (Table 1). The mean age was 70.4 years (*SD* = 12.2) and 9 patients were smokers. Each patient had one implant explanted. The mean implant diameter was 3.6 (*SD* = 0.3) and mean implant length 11.3 mm (*SD* = 1.7). The majority of the implants explanted were in the anterior upper jaw (68%). The mean radiographic exposed implant length (A‐C) was 6.1 mm (*SD* = 1.5) and the mean radiographic intact implant socket length (D‐C) was 5.2 mm (*SD* = 0.9). The mean percentage of bone loss with respect to the length of the implants was 53.6% (*SD* = 7.2). The mean intrabony defect depth (B‐C) was 4.8 mm (*SD* = 1.6) and the mean alveolar bone crest level B‐D was 9.1 mm (*SD* = 2.1).

**Table 1:** Baseline data from the 31 patients and implants.

**Number of patients**	31
Age, mean (*SD*)	70.4 (12.2)
Female, number and (%)	20 (64.5%)
Smokers, number and (%)	9 (29%)
**Number of implants at baseline**	31
Exposed implant length (A‐C) mm, mean (*SD*)	6.1 (1.5)
Intact implant socket length (D‐C) mm, mean (*SD*)	5.2 (0.9)
Percentage of bone loss/implant length, mean (*SD*)	53.6 (7.2)
Intrabony defect depth (B‐C) mm, mean (*SD*)	4.8 (1.6)
Implant length (mm), mean (*SD*)	11.3 (1.7)
Implant diameter (mm), mean (*SD*)	3.6 (0.3)
Implant position	
Upper Jaw	
Anterior	16
Premolar	5
Molar	0
Lower Jaw	
Anterior	2
Premolar	8
Molar	0

### 3.2 Six‐month evaluation

At 6 months following implants removal, the radiographic measurements showed a decrease in the mean intrabony defect depth (B‐C) of 1.6 mm (*SD* = 1.5) as a result of some bone healing from the bottom of the bone defect (i.e., D‐C change) of 0.8 mm (*SD* = 1.1) and marginal resorption of the alveolar bone crest (i.e., B‐D change) of 0.8 mm (*SD* = 1.3) (Table 2). The results of the Wilcoxon signed‐rank test indicted a significant difference between each of these measurement indicators and the baseline value (Table 2). The multilinear regression analysis revealed that the decrease of the bone level (B‐D) between Baseline and 6 months was affected by the absence of alveolar buccal bone (unstandardized regression coefficient [*B*] = −1.235, *p* = .011) and with the depth of the defect (B‐C), *B* = −0.229, *p* = .049. Healing from the bottom of the bone defect (D‐C change), was positively correlated with the depth of the alveolar bone defect B‐C (*B* = 0.208, *p* = .033), with the percentage of bone loss/implant length (*B* = 0.0058, *p* = .017) and negatively correlated with the absence of the alveolar buccal bone (*B* = −1.288, *p* < .01) (Table 3).

**Table 2:** Baseline and 6‐month data and statistical analysis of radiographical and clinical measurement changes.

Total number of implants: 31	Mean	Standard deviation	Median	Minimum	Maximum	Interquartile range
Intrabony defect depth B‐C (Bl)	4.8	1.6	5.0	1.5	7.4	2.5
Bone crest level B‐D (Bl)	9.9	2.0	10.0	5.5	13.0	3.2
Intact implant socket depth D‐C (Bl)	5.2	0.9	5.3	4.0	8.3	1.6
Intrabony defect depth B‐C (6 months)	3.2	1.3	3.5	1.0	5.6	1.7
Bone crest level B‐D (6 months)	9.1	2.1	9.2	4.0	14.0	2.4
Intact implant socket depth C‐D (6 months)	6.0	1.3	5.8	3.6	9.0	1.6
Mucosa height change	−0.4	0.7	0.00	−2	1	1
Mucosa width change	−0.7	0.8	−1.0	−2	1	1
BD_change (“6 month”‐“BL”)	−0.8	1.3	−0.4	−4.30	1.00	1.1
DC_change (“6 month”‐“BL”)	0.8	1.1	0.7	−2.20	4.20	1.5
BC_change (“6 month”‐“BL”)	−1.6	1.5	−1.3	−4.80	1.30	2.4

Wilcoxon signed‐rank test	*p*‐value					
BD (“BL” vs. “6 month”)	.001					
CD (“BL” vs. “6 month”)	.001					
BC (“BL” vs. “6 month”)	.001					

**Table 3:** Generalized estimating equations (multilevel linear regression analyses) with B‐D and D‐C changes as dependent variables and % of bone loss/implant length, implant width, presence/absence of buccal bone, bony defect, as independent variables.

Dependent variable: B–D change Bl—6 months				
Indipendent variables	Coefficient	95% confidence interval		
		Lower	Upper	*p*‐value
% Bone loss/length	0.036	−0.033	0.105	.310
Implant diameter	−0.313	−1.427	0.801	.581
Buccal bone (0: presence, 1: absence)	−1.235	−2.191	−0.278	.011
Bony defect B‐C Bl	−0.229	−0.457	−0.001	.049

**Dependent variable: C–D change Bl**—**6 months**				
**Independent variables**	**Coefficient**	**95% confidence interval**		
		**Lower**	**Upper**	***p*‐value**
% Bone loss/length	0.058	0.011	0.106	.017
Implant diameter	−0.083	−1.268	1.101	.890
Buccal bone (0: presence, 1: absence)	−1.288	−2.078	−0.498	.001
Intrabony defect depth B‐C Bl	0.208	0.017	0.399	.033

A reduction of 0.4 mm of the mucosa height was recorded (*SD* = 0.7) concomitant with a decrease of 0.7 mm of the buccal‐lingual/palatal mucosa width (*SD* = 0.8). The probability to have a decrease in mucosa height and width of ≥2 mm was associated with the absence of alveolar buccal bone (*p* = .007 and *p* = .001, respectively; Fisher's exact probability test).

## 4 DISCUSSION

**Figure 4:**
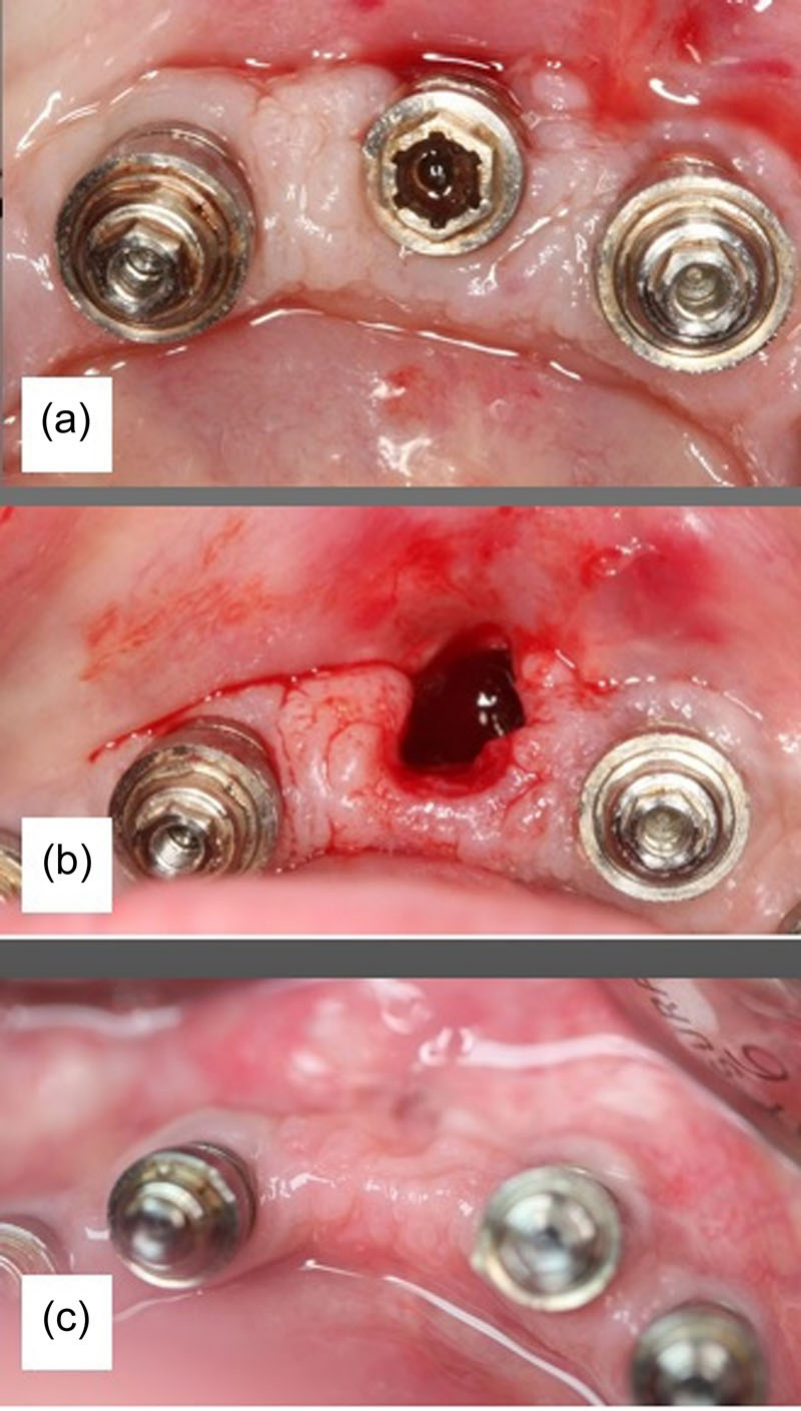
(a) Implant before explantation, (b) Occlusal view of the alveolus following implant explantation; no buccal bone was present, (c) Alveolar ridge 6 months following implant explantation: it is possible to observe the pronounced reduction in the width of the soft tissue contour.

While the healing process of alveolar bone following tooth extraction has been extensively described in the literature (Araujo et al., 2015), this is not the case for the healing following implant explantation. Therefore, the present study intended to explore the hard and soft tissue alterations following explantation of implants with persisting peri‐implant disease despite treatment. The alveolar bone at an implant extraction socket, explanted due to peri‐implants, can be divided in two compartments: an apical, where the bone walls were still in intimate contact with the implant (i.e., the intact implant socket) and a coronal portion, where any remaining (non‐resorbed) bone walls were at a distance from the implant (i.e., the peri‐implant bone defect). Based on the radiographical analysis herein, following implant explantation, the apical compartment of the implant alveolus (D‐C) seems to heal completely, and some additional bone gain occurs in a coronal direction at the bottom of the peri‐implantitis bone defect. Bone gain was positively correlated with the depth of the intrabony defect (B‐C) and with the presence of alveolar buccal bone, i.e., deeper intrabony defects and more intact sockets showed more bone gain comparing to shallower intrabony defects and less intact sockets; thus, this pattern of healing may not apply in purely horizontal peri‐implantitis bone defects. In the coronal compartment, however, the implant alveolus exhibits some resorption of the bone crest, which is correlated with the depth of the depth of the peri‐implantitis bone defect (B‐C) and with the absence of buccal bone (i.e., deeper intrabony defects and less intact sockets, showed more crestal bone resorption comparing to shallower intrabony defect and more intact sockets). As consequence of the hard tissue remodeling, a decrease in the height and buccal‐lingual/palatal width of the overlying mucosa was also observed. This soft tissue (contour) remodeling was more pronounced in width than in height, and more pronounced in the absence of alveolar buccal bone (Figure 4).

The healing pattern observed in the implant alveolus after explantation resembled only partly that in a tooth extraction socket. Specifically, only the slight decrease (i.e., 0.8 mm, on average) of the radiographical alveolar bone crest level following implant explantation is comparable with the values reported by Lekovic et al., 1998 and Serino et al., 2003 regarding alveolar crest resorption following tooth extraction. In contrast, bone healing in the remaining implant extraction socket was limited and did not follow the pattern of healing in a tooth extraction socket, which becomes largely filled out with bone (Araujo & Lindhe, 2005; Trombelli et al., 2008). Herein, bone healing only slightly exceeded (i.e., 0.8 mm, on average) the apical compartment of the implant alveolus (i.e., the intact implant socket).

Thus, bone healing basically regarded the narrow cylindrical defect at the bottom of the implant socket, corresponding to the implant diameter (herein being 3.3–4.2 mm), and representing the part of the implant that was still osseointegrated. In this context, the minimal traumatic explantation technique used herein minimized the damage at the peri‐implant bone walls, and probably contributed to a fast and uneventful healing. Indeed, assessments of human histologies have shown that healing of a tooth extraction socket starts from the apical and lateral residual bone walls (Trombelli et al., 2008) and consists mainly of mineralized new bone in the apical aspect, while the coronal aspect is less mineralized up to 3–6 months of healing (Serino et al., 2003, 2007). Thus, it may be that the tissue filling in the coronal aspect of the implant alveolus consisted of newly formed woven bone, that it was simply not yet mineralized enough at 6 months after explanation to be visualized in the radiographs. Nevertheless, it must be also mentioned that based on human bone core biopsies harvested from extraction sockets of periodontitis involved and non‐involved teeth (Ahn & Shin, 2008), new bone formation was markedly delayed within the healing extraction sockets of periodontally involved teeth compared with those of teeth extracted due to other reasons. Thus, the possibility of delayed and/or compromised healing in the alveola of implants explanted due to peri‐implantitis cannot be excluded. Such impaired healing is at least partly due to the often‐compromised bone architecture (i.e., reduced number of bone walls, large defects) in advanced peri‐implantitis defects (Wehner et al., 2020) and thereby due to the reduced tissue resources contributing to healing, compared to intact alveoli. Larger bone resorption was indeed noted herein at sites where the buccal bone was not present. At implants, in contrast to teeth where the buccal bone is thinner than the palatal/lingual bone (Januario et al., 2011), the thickness of the buccal and palatal/lingual bone—and thus how much of it may remain in the event of peri‐implantitis—depends by the implant diameter and location/inclination with respect to the width of the alveolar crest at the timepoint of implant installation. When the buccal alveolar bone is absent after tooth extraction, the placement of filling graft materials in the postextraction alveolus (with or without membranes) seems to diminish alveolar ridge reduction (Park et al., 2020). If this is the case following implant explantation, it remains to be evaluated.

Regarding the changes in the soft tissues, a larger change was observed in the buccal‐lingual/palatal horizontal dimension comparing to the vertical one, i.e., on average 0.7 versus 0.4 mm, respectively, and was more pronounced in the absence of buccal bone. This observation follows the known pattern of dimensional changes in the alveolar ridge after tooth extraction, however, the changes were rather smaller in magnitude that what reported for tooth sites. Based on a recent meta‐analysis, after tooth extraction, the average mean horizontal and vertical mid‐buccal reduction in the alveolar ridge is on average 2.73 and 1.71 mm, respectively. This difference in the dimensional changes after tooth extraction versus implant explantation, are most likely explained by the morphological differences in the tooth and implant socket, with the former being largely composed of cortical and bundle bone, that experience extended resorption during post‐extraction healing (Araújo et al., 2015).

This study comes by the nature of its retrospective design and the clinical settings with inevitable drawbacks. For example, (a) no information about the thickness of the buccal wall at implant installation/restoration was available herein, (b) the clinical measurements were done with a periodontal probe, a relatively crude method, and (c) the radiographs although taken using a parallel technique with film‐holder, they were not standardized and may have influenced the precision of the measurements. Furthermore, the observations of this study should be applicable only to implants affected by peri‐implantitis, with limited residual osseointegration (all implants presented >50% bone loss), meaning that a minimal traumatic technique (i.e., the torque needed to unscrew the implants was low), could be employed. Thus, these results are likely not applicable for explantation of fully osseointegrated implants and for other explanation techniques (i.e., trephine bur, piezo‐surgery). In this context, the possible impact of the presence or absence of neighboring teeth, as well as of the distance to‐ and health and/or bone level status of neighboring implants was not possible to assess due to the limited number of included cases.

## 5 CONCLUSION

The results of the present explorative study indicate that there is a decrease in the height and width of the alveolar soft and hard tissues following explantation of peri‐implantitis affected implants, and these changes were more pronounced in the absence of the buccal bone wall. Nevertheless, the apical portion of the implant alveolus (the intact implant socket) tends to heal with no further bone loss.

## AUTHOR CONTRIBUTIONS

**Giovanni Serino**: Study design; data collections; analysis; interpretation; manuscript writing; conclusions. **Masahiro Wada**: Radiographic examinations; data analysis; interpretation. **Tomoaki Mameno**: Radiographic examination; statistical analysis; data analysis; interpretation. **Andreas Stavropoulos**: Manuscript critical revision; interpretation; writing.

## CONFLICT OF INTEREST STATEMENT

The authors declare no conflicts of interest.

## Data Availability

Data that support the findings of this study are available upon reasonable request from the corresponding author. The data are not publicly available due to privacy or ethical restrictions.
